# Dietary strawberry seed oil affects metabolite formation in the distal intestine and ameliorates lipid metabolism in rats fed an obesogenic diet

**DOI:** 10.3402/fnr.v59.26104

**Published:** 2015-01-29

**Authors:** Adam Jurgoński, Bartosz Fotschki, Jerzy Juśkiewicz

**Affiliations:** Division of Food Science, Institute of Animal Reproduction and Food Research, Polish Academy of Sciences, Olsztyn, Poland

**Keywords:** α-linolenic acid, butyrate, cecum, linoleic acid, strawberry seed oil, triglycerides

## Abstract

**Objective:**

To answer the question whether dietary strawberry seed oil rich in α-linolenic acid and linoleic acid (29.3 and 47.2% of total fatty acids, respectively) can beneficially affect disorders induced by the consumption of an obesogenic diet.

**Design:**

Thirty-two male Wistar rats were randomly assigned to four groups of eight animals each and fed with a basal or obesogenic (high in fat and low in fiber) diet that contained either strawberry seed oil or an edible rapeseed oil. A two-way analysis of variance was then applied to assess the effects of diet and oil and the interaction between them.

**Results:**

After 8 weeks of feeding, the obesogenic diet increased the body weight and the liver mass and fat content, whereas decreased the cecal acetate and butyrate concentration. This diet also altered the plasma lipid profile and decreased the liver sterol regulatory element-binding protein 1c (SREBP-1c) content. However, the lowest liver SREBP-1c content was observed in rats fed an obesogenic diet containing strawberry seed oil. Moreover, dietary strawberry seed oil decreased the cecal short-chain fatty acid concentrations (acetate, propionate, and butyrate) regardless of the diet type, whereas the cecal *β*-glucuronidase activity was considerably increased only in rats fed an obesogenic diet containing strawberry seed oil. Dietary strawberry seed oil also lowered the liver fat content, the plasma triglyceride level and the atherogenic index of plasma.

**Conclusions:**

Strawberry seed oil has a potent lipid-lowering activity but can unfavorably affect microbial metabolism in the distal intestine. The observed effects are partly due to the synergistic action of the oil and the obesogenic diet.

An energy-dense dietary pattern rich in fat and refined carbohydrates and low in fiber is one of the most important causes for obesity (1). In obese subjects, an aberrant gut microbiota with impaired metabolic activity, especially carbohydrate fermentation and bile acid metabolism, has been recognized (2, 3). Changes in gut microbial metabolism seem to be also involved in the development of colorectal cancer which is closely associated both with obesity and with the aforementioned dietary pattern (4). Altered metabolism of bile acids can lead to the formation of potentially carcinogenic *N*-nitroso compounds, whereas limited fermentation of dietary fiber and resistant starch can reduce short-chain fatty acid (SCFA) production in the distal intestine, including butyrate that promotes a normal phenotype for colonocytes and protects against damage of their DNA (4, 5). Moreover, there is a growing body of evidence suggesting that the type of dietary fat may also be an important factor involved in colon carcinogenesis. Epidemiological and experimental studies have shown that a high intake of n-6 polyunsaturated fatty acids (PUFAs) may promote, whereas n-3 PUFAs can suppress carcinogenesis; however, the exact mechanism responsible for these effects is unclear and may involve eicosanoid production, inflammation, and oxidative stress (6, 7).

Fat is a crucial macronutrient that affects the development of cardiovascular disease (CVD), which in turn is the most prevalent complication of obesity. The recommended nutritional strategy for CVD prevention is increasing consumption of unsaturated fatty acids, including PUFAs, at the expense of saturated fatty acids (SFAs). Numerous mechanisms whereby dietary PUFAs can ameliorate obesity-related disorders and reduce CVD risk have been identified, including beneficial effects on adipocyte-derived hormones, a low-grade inflammation, blood lipids, blood pressure, and vasodilation (8, 9). These effects especially involve n-3 PUFAs, namely eicosapentaenoic acid and docosahexaenoic acid, found in fish oils (10). In contrast, for the n-3 α-linolenic acid, which is present as a relatively small proportion of common edible plant oils, the relation to cardiovascular health is less clear (11, 12).

Strawberry oil, produced from the seeds that are by-products of the industrial processing of strawberries, is an example of unconventional plant oils which can be considered as new food stuffs. This oil has an interesting fatty acid profile with high proportion of PUFAs (78% of the oil), including a considerable proportion of α-linolenic acid (36% of the oil) and a favorable low n-6:n-3 PUFA ratio (1.16) (13). Moreover, strawberry seed oil has been reported to contain significant amounts of antioxidants, including potentially bioactive phenolic compounds (14, 15).

In the current study, given the high nutritional value of strawberry seed oil, it was hypothesized that this oil can beneficially affect disorders induced by the consumption of an obesogenic diet. A biological response to dietary strawberry seed oil, including its effects on a local metabolite formation in the distal intestine, blood lipid profile, markers of inflammation, and liver function, was determined in rats. An obesogenic diet with a relatively low fiber content and a high fat content was used to obtain an animal model of metabolic disorders related to obesity and its complications.

## Materials and methods

### Dietary fats and their analysis

An edible refined rapeseed oil (Canola, ZT Kruszwica SA, Kruszwica, Poland) and pork lard (Animex Foods Ltd., Ostróda, Poland) were purchased in a local supermarket. Unrefined cold-pressed and filtered strawberry seed oil was purchased from Greenaction (Kielce, Poland). The fatty acid profile of dietary fats was determined using a gas chromatography method described elsewhere (16). The fatty acid profile of lard was as follows: C14:0 (1.6%), C16:0 (29.3%), C18:0 (0.5%), C18:1 n-9 (58.1%), C18:2 n-6 (8.9%), C18:3 n-3 (0.6%), and C20:0 (0.9%). The fatty acid profile of rapeseed oil and strawberry seed oil is given in Table 1.

**Table 1:** Fatty acid profile of the dietary oils (%)

	Rapeseed oil	Strawberry seed oil
C16:0	4.58±0.032	4.70±0.046
C18:0	0.544±0.014	0.586±0.028
C18:1 n-9	63.5±0.17	16.9±0.04
C18:2 n-6	20.1±0.13	47.2±0.04
C18:3 n-3	9.02±0.031	29.3±0.07
C20:0	2.20±0.030	1.29±0.007

The results are presented as the mean±SD (*n*=3).

### Nutritional experiment

The nutritional experiment was performed on 32 male Wistar rats weighing 127.2±5.46 g on average, randomly assigned to one of four groups of eight rats each. The animals were maintained individually in metabolic cages under a stable temperature (21–22°C), a 12-h light:12-h dark cycle and a ventilation rate of 15 air changes per hour. For 8 weeks, the rats had free access to tap water and semi-purified diets (details in Table 2), which were freshly prepared at weekly intervals and stored in a freezer in hermetic containers. The diets were modifications of a casein diet for laboratory rodents recommended by the American Institute of Nutrition (17). Two groups of rats were fed with a basal diet containing either 7% of rapeseed oil (group B-RO) or 7% of strawberry seed oil (group B-SO) as the sole source of fat. The other two groups were fed with an obesogenic diet, which had the same proportion of rapeseed oil (group O-RO) or strawberry seed oil (group O-SO), but was additionally supplemented with lard and cholesterol (14 and 0.5% of the diet, respectively,) and had a relatively low cellulose content (2% of the diet). The use of rats was conducted in compliance with European guidelines for the care and use of laboratory animals, and the animal protocol was approved by the Local Institutional Animal Care and Use Committee (Olsztyn, Poland).

**Table 2:** Composition of the group-specific diets

	Group			
	
Ingredient (%)	B-RO	B-SO	O-RO	O-SO
Casein	20	20	20	20
DL-methionine	0.3	0.3	0.3	0.3
Lard^a^	–	–	14	14
Rapeseed oil (Canola)^b^	7	–	7	–
Strawberry seed oil^b^	–	7	–	7
Cholesterol	–	–	0.5	0.5
α-Cellulose	5	5	2	2
Sucrose	10	10	10	10
Corn starch	53.0	53.0	41.5	41.5
Mineral mix^c^	3.5	3.5	3.5	3.5
Vitamin mix^c^	1	1	1	1
Choline chloride	0.2	0.2	0.2	0.2
Calculated total fat content (%)	7.3	7.3	21.3	21.3
SFAs	0.51	0.46	5.04	4.98
MUFAs	4.45	1.18	12.58	9.32
PUFAs	2.04	5.36	3.37	6.69
n-6	1.41	3.30	2.65	4.55
n-3	0.63	2.05	0.71	2.14
Unidentified^d^	0.34	0.34	0.33	0.33
Energy density (kcal/g)	3.64	3.64	4.50	4.50

SFAs=saturated fatty acids; MUFAs=monounsaturated fatty acids; PUFAs=polyunsaturated fatty acids.

a Fatty acid profile: C14:0 (1.6%), C16:0 (29.3%), C18:0 (0.5%), C18:1 n-9 (58.1%), C18:2 n-6 (8.9%), C18:3 n-3 (0.6%) and C20:0 (0.9%).

b Fatty acid profile in Table 1.

c Recommended for the AIN-93G diet (17).

d From casein and corn starch preparations.

### Sample collection and analysis

Upon termination of the experiment, the rats were weighed and anesthetized with sodium pentobarbital (50 mg/kg body weight). The fat and lean body mass was then determined by time-domain nuclear magnetic resonance using the minispec LF 90II analyzer (Bruker, Karlsruhe, Germany). The minispec transmits various radio frequency pulse sequences into soft tissues to re-orient the nuclear magnetic spins of the hydrogen and then detects radio frequency signals generated by the hydrogen spins from these tissues. The contrast in relaxation times of the hydrogen spins found between adipose tissue and water-rich tissues is used to estimate fat and lean body mass.

Blood samples were collected from the caudal vein into EDTA tubes, centrifuged for 15 min at 380×*g*, and the obtained plasma was then stored at −20°C until analysis. The plasma triglyceride (TG) concentration and the plasma concentration of total cholesterol (TC) and its HDL fraction (HDL-C) were determined using reagents from Alpha Diagnostics Ltd. (Warsaw, Poland). The plasma non-HDL cholesterol (non-HDL-C) was calculated as the difference between TC (mmol/L) and HDL-C (mmol/L). The atherogenic index (AI) of plasma was calculated as previously described (18) using the following formula: log(TGs (mmol/L)/HDL-C (mmol/L)). The plasma aspartate transaminase (AST) activity and the plasma alanine transaminase (ALT) activity were estimated using kits from Alpha Diagnostics Ltd. (Warsaw, Poland). The plasma C-reactive protein (CRP) concentration was measured using an enzyme immunoassay kit containing an antibody specific for rat CRP (Cusabio, Wuhan, China).

The cecum was removed and weighed, and samples of the cecal digesta were collected, fractions of which were stored at −70°C. In the fresh cecal digesta, the pH was measured using the SevenMulti pH meter (Mettler-Toledo, Warsaw, Poland), whereas the dry matter was examined after drying at 105°C. The SCFA concentration was determined in the stored cecal digesta using a gas chromatography method described elsewhere (16). The *β*-glucuronidase activity in the stored cecal digesta was measured spectrophotometrically using a method described elsewhere (19), and was expressed as mmol of product formed per hour per g of digesta.

The liver and epididymal fat were removed and weighed, and then the liver fat mass was determined by the minispec LF 90II analyzer (Bruker, Karlsruhe, Germany). After storage of the liver at −20°C, the sterol regulatory element-binding protein 1c (SREBP-1c) content was determined using a validated rat enzyme immunoassay kit (Cusabio, Wuhan, China).

### Statistical analysis

The results are expressed as the mean±the standard error of the mean (SEM), except for the fatty acid content of dietary oils which is expressed as the mean and the standard deviation (SD) of the mean. A two-way analysis of variance (ANOVA) was used to determine the effects of diet (basal or obesogenic, D) and oil (rapeseed or strawberry seed, O) and the interaction between these two factors (D×O). If the analysis revealed a significant interaction (*P*≤0.05), the differences among the treatment groups were then determined with Duncan's *post hoc* test at *P*≤0.05. The statistical analysis was performed using STATISTICA software, version 8.0 (StatSoft Corp., Krakow, Poland).

## Results

The detailed fatty acid profile of the oils used as the sources of fat in the experimental diets are shown in Table 1. The rapeseed oil used in the diets of the B-RO and O-RO groups was rich in oleic acid (63.5%); linoleic acid (20.1%); and, to a lesser extent, α-linolenic acid (9.02%). The strawberry seed oil used in the diets of the B-SO and O-SO groups was especially rich in linoleic acid (47.2%) and α-linolenic acid (29.3%), but the oleic acid proportion was considerable as well (16.9%).

After the 8-week feeding period, for the obesogenic feeding regimen, the calorie intake and the body weight gain were increased (*P*<0.05, Table 3). The total fat and lean body mass were not affected by either the diet type or the oil type (*P*>0.05), whereas the proportion of epididymal fat was increased by the obesogenic diet (*P*<0.05).

**Table 3:** Calorie intake, body weight gain, and the proportion of body fat and lean mass in rats after 8 weeks of feeding with experimental diets

		Body			
		
	Calorie intake^a^	Weight gain (g)	Fat mass (%)	Epididymal fat mass (%)	Lean mass (%)
Group					
B-RO	61.1±1.79	212±9.5	37.0±0.81	3.00±0.240	35.2±1.16
B-SO	59.3±1.35	208±7.3	38.3±0.86	2.78±0.116	33.9±1.07
O-RO	67.5±1.89	253±10.7	40.4±1.29	3.86±0.364	27.9±1.89
O-SO	67.3±1.56	243±10.6	39.6±0.87	3.56±0.203	30.4±1.16
Diet (D)					
Basal	60.1±1.08	210±5.7	37.7±0.60	2.89±0.126	34.5±0.78
Obesogenic	67.4±1.17	248±7.4	40.0±0.74	3.70±0.198	29.2±1.09
*P*	0.032	0.038	0.133	0.029	0.058
Oil (O)					
Rapeseed	64.3±1.54	233±8.9	38.7±0.87	3.43±0.241	31.5±1.47
Strawberry seed	63.3±1.44	225±7.6	38.9±0.62	3.17±0.151	32.1±0.89
*P*	0.988	0.440	0.644	0.965	0.961
Interaction (D×O)					
*P*	0.774	0.730	0.788	0.817	0.550

Groups B-RO and B-SO were fed with a basal diet containing rapeseed oil or strawberry seed oil, respectively, as the source of fat. Groups O-RO and O-SO were fed with an obesogenic diet containing rapeseed oil or strawberry seed oil, respectively.

The results are presented as the mean±SEM.

a kcal/day.

Basic indices of the cecal digesta and the ammonia and SCFA concentration in the cecal digesta of rats are shown in Table 4. The cecal tissue mass (data not shown) and the relative digesta mass were comparable among all groups (*P*>0.05). However, the digesta dry matter was decreased by the obesogenic feeding regimen (*P*<0.05). The digesta pH value was not increased by dietary factors, including strawberry seed oil (*P*=0.058). The total SCFA concentration and nearly all particular SCFA concentrations were affected by both experimental factors; strawberry seed oil decreased the acetate, propionate, butyrate, and total SCFA concentration (*P*<0.05), and the obesogenic diet also decreased the total and each particular SCFA concentration (*P*<0.05), except for the propionate concentration which was not different compared to the basal diet (*P*>0.05). The SCFA profile was only affected by the type of diet; the obesogenic diet increased the acetate and propionate proportions (*P*<0.05), whereas the butyrate proportion was decreased (*P*<0.001).

**Table 4:** Basic indices and ammonia and short-chain fatty acid (SCFA) concentrations in the cecal digesta of rats

					SCFA concentration (µmol/g)				SCFA profile (%)		
						
	Mass^a^	Dry matter (%)	pH	NH_3_ (mg/g)	C2	C3	C4	Total	C2	C3	C4
Group											
B-RO	3.63±0.406	24.3±0.64	7.21±0.116	0.316±0.034	123.4±6.39	20.5±0.91	28.3±2.96	181±8.7	68.3±1.38	11.5±0.64	15.5±1.24
B-SO	3.03±0.222	25.5±0.28	7.47±0.087	0.307±0.014	85.5±2.38	15.8±1.00	18.3±1.21	127±4.2	67.2±0.82	12.4±0.51	14.3±0.50
O-RO	3.04±0.276	20.5±0.61	7.30±0.062	0.407±0.042	98.8±7.36	20.8±1.67	11.2±0.66	139±10.2	71.3±0.47	15.0±0.24	8.2±0.37
O-SO	3.18±0.360	24.9±0.97	7.41±0.079	0.346±0.024	81.3±6.23	17.5±0.78	9.1±0.92	114±8.01	70.9±0.93	15.5±0.63	7.9±0.43
Diet (D)											
Basal	3.31±0.229	24.9±0.40	7.35±0.077	0.311±0.017	103.2±5.94	18.0±0.91	23.0±1.98	152±8.40	67.8±0.76	12.0±0.41	14.9±0.63
Obesogenic	3.11±0.224	22.9±0.82	7.36±0.052	0.374±0.024	89.4±5.16	19.1±0.96	10.1±0.63	126±6.96	71.1±0.53	15.3±0.35	8.0±0.28
*P*	0.157	0.042	0.762	0.271	0.033	0.969	0.000	0.013	0.046	0.000	0.000
Oil (O)											
Rapeseed	3.33±0.249	22.4±0.67	7.25±0.064	0.361±0.029	111.1±5.79	20.7±0.91	19.7±2.78	160±8.70	69.8±0.81	13.2±0.59	11.9±1.19
Strawberry seed	3.10±0.205	25.2±0.56	7.44±0.057	0.327±0.014	83.4±3.27	16.7±0.65	13.7±1.40	121±4.7	69.1±0.76	14.0±0.56	11.1±0.89
*P*	0.237	0.212	0.058	0.929	0.009	0.048	0.016	0.007	0.266	0.120	0.286
Interaction (D×O)											
*P*	0.421	0.346	0.740	0.300	0.423	0.524	0.647	0.478	0.191	0.352	0.391

Groups B-RO and B-SO were fed with a basal diet containing rapeseed oil or strawberry seed oil, respectively, as the source of fat. Groups O-RO and O-SO were fed with an obesogenic diet containing rapeseed oil or strawberry seed oil, respectively. C2, acetate; C3, propionate; C4, butyrate. The results are presented as the mean±SEM.

a g/g cecal tissue.

**Fig. 1:**
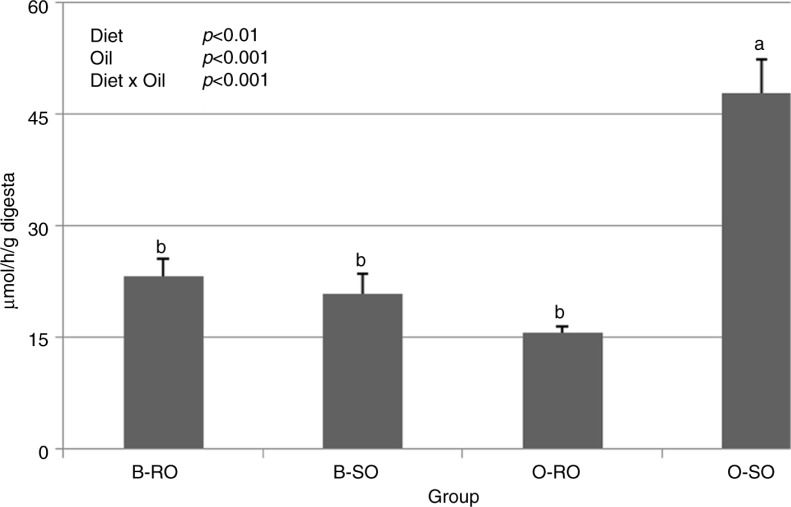
Microbial *β*-glucuronidase activity in the cecal digesta of rats. Groups B-RO and B-SO were fed with a basal diet containing rapeseed oil or strawberry seed oil, respectively, as the source of fat. Groups O-RO and O-SO were fed with an obesogenic diet containing rapeseed oil or strawberry seed oil, respectively. The results are presented as the mean±SEM. Mean values with unlike letters (a, b) were significantly different in Duncan's *post hoc* test (*P*<0.05).

The *β*-glucuronidase activity in the cecal digesta was affected by both experimental factors (D, *P*<0.01 and O, *P*<0.001) and their interaction (D×O, *P*<0.001), thus the activity was higher in group O-SO than in the other groups (*P*≤0.05; Fig. 1).

**Fig. 2:**
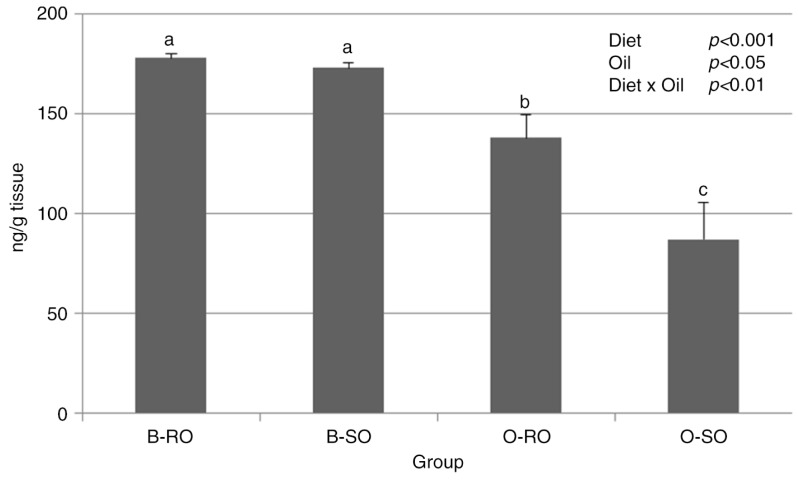
SREBP-1c content in the liver of rats. Groups B-RO and B-SO were fed with a basal diet containing rapeseed oil or strawberry seed oil, respectively, as the source of fat. Groups O-RO and O-SO were fed with an obesogenic diet containing rapeseed oil or strawberry seed oil, respectively. The results are presented as the mean±SEM. Mean values with unlike letters (a, b, c) were significantly different in Duncan's *post hoc* test (*P*<0.05).

Both factors had an effect on basic liver indices (Table 5). The obesogenic dietary regimen increased the liver mass, relative to the body weight, and the percentage content of fat in the liver (*P*<0.001). Dietary strawberry seed oil decreased the percentage content of fat in the liver compared to dietary rapeseed oil (*P*<0.05), but the relative liver mass was not changed. Furthermore, the liver SREBP-1c content was affected by both the type of diet and the type of oil (*P*<0.001 and *P*<0.05, respectively), and an interaction effect between these factors was also observed (D×O, *P*<0.01; Fig. 2). The highest content was found in groups B-RO and B-SO, whereas the content was significantly lower in group O-RO, and the lowest SREBP-1c content was in group O-SO (*P*≤0.05).

**Table 5:** Markers of liver function and inflammation in rats after 8 weeks of feeding with experimental diets

	Liver		Plasma		
		
	Mass^a^	Fat mass (%)	ALT (U/L)	AST (U/L)	CRP (ng/mL)
Group					
B-RO	3.64±0.138	25.9±2.21	20.9±1.13	107±1.8	0.198±0.041
B-SO	3.25±0.145	21.3±2.01	21.1±1.00	94±4.2	0.144±0.041
O-RO	4.59±0.141	50.8±2.96	33.9±3.61	137±15.9	0.538±0.104
O-SO	4.26±0.092	42.5±3.00	39.5±4.38	138±12.7	0.303±0.107
Diet (D)					
Basal	3.43±0.110	23.5±1.56	21.0±0.72	100±3.0	0.171±0.029
Obesogenic	4.41±0.091	46.3±2.32	36.9±2.89	137±9.58	0.421±0.080
*P*	0.000	0.000	0.005	0.076	0.016
Oil (O)					
Rapeseed	4.11±0.162	38.3±3.87	27.4±2.67	122±8.9	0.368±0.074
Strawberry seed	3.76±0.154	31.9±3.24	30.3±3.35	116±8.8	0.223±0.060
*P*	0.104	0.019	0.220	0.673	0.086
Interaction (D×O)					
*P*	0.986	0.405	0.133	0.400	0.532

Groups B-RO and B-SO were fed with a basal diet containing rapeseed oil or strawberry seed oil, respectively, as the source of fat. Groups O-RO and O-SO were fed with an obesogenic diet containing rapeseed oil or strawberry seed oil, respectively.

ALT=alanine transaminase; AST=aspartate transaminase; CRP=C-reactive protein.

The results are presented as the mean±SEM.

a g/100 g body weight.

The plasma ALT activity and the plasma CRP concentration were increased by the obesogenic dietary regimen (*P*<0.05; Table 5). The type of oil had no effect on these inflammation markers (*P*>0.05). However, the plasma CRP concentration tended to be lower in the strawberry seed oil-fed groups (*P*=0.086). Moreover, the plasma concentration of tumor necrosis factor alpha was comparable among all dietary groups (data not shown).

The obesogenic diet substantially changed the plasma lipid profile; however, the AI of plasma was comparable with the basal dietary regimen. The obesogenic diet decreased the TG and HDL-C concentration (*P*<0.01) and increased the TC and non-HDL-C concentration (*P*≤0.001, Table 6). Furthermore, the TG concentration was affected by the type of dietary oil (*P*<0.001), and an interaction between the type of diet and the type of dietary oil was also noted (D×O, *P*<0.05). Group O-SO had the lowest TG concentration, whereas the highest TG concentration was observed in group B-RO (*P*≤0.05). The TG concentration in groups B-SO and O-RO was on a moderate level and differed significantly from those of the B-RO and O-SO groups (*P*≤0.05). Moreover, the AI of plasma was decreased by strawberry seed oil (*P*<0.01; Table 6).

**Table 6:** Lipid profile and atherogenic index of rat plasma

	TGs (mmol/L)	TC (mmol/L)	HDL-C (mmol/L)	Non-HDL-C (mmol/L)	AI*
Group					
B-RO	2.25±0.224^a^	1.90±0.181	1.37±0.164	0.52±0.064	0.215±0.076
B-SO	1.07±0.165^b^	1.92±0.138	1.35±0.110	0.56±0.055	−0.124±0.068
O-RO	1.06±0.152^b^	2.63±0.142	0.95±0.060	1.68±0.154	0.029±0.078
O-SO	0.83±0.119^c^	2.52±0.126	0.84±0.043	1.68±0.093	−0.033±0.064
Diet (D)					
Basal	1.61±0.214	1.91±0.107	1.36±0.092	0.55±0.041	0.033±0.069
Obesogenic	0.93±0.097	2.57±0.092	0.89±0.038	1.68±0.083	−0.004±0.048
*P*	0.001	0.001	0.004	0.000	0.151
Oil (O)					
Rapeseed	1.65±0.221	2.27±0.157	1.16±0.104	1.10±0.192	0.122±0.059
Strawberry seed	0.95±0.104	2.22±0.122	1.10±0.091	1.12±0.163	−0.078±0.047
*P*	0.000	0.929	0.773	0.949	0.002
Interaction (D×O)					
*P*	0.021	0.802	0.347	0.796	0.183

Groups B-RO and B-SO were fed with a basal diet containing rapeseed oil or strawberry seed oil, respectively, as the source of fat. Groups O-RO and O-SO were fed with an obesogenic diet containing rapeseed oil or strawberry seed oil, respectively.

AI=atherogenic index; HDL-C=HDL-cholesterol; non-HDL-C=the difference between TC and HDL-C; TC=total cholesterol; TGs=triglycerides.

The results are presented as the mean±SEM.

a, b, c Mean values within a column with unlike superscript letters (a, b, c) were significantly different in Duncan's *post hoc* test (*P*<0.05).

* log(TG/HDL-C).

## Discussion

The fatty acid profile of the tested strawberry seed oil was slightly different from the profile described in previous reports (13, 20). Compared with the results obtained by Van Hoed et al. (13), in the current study, the linoleic acid content was higher by approximately five percentage points, whereas the α-linolenic acid content was lower by approximately seven percentage points (Table 1). The observed disparities can be a consequence of the different growth conditions of strawberries as it is the case for oily plants (21). Nevertheless, the strawberry seed oil was still a rich source of α-linolenic acid (29.3%) compared to a much more common plant oils, like rapeseed oil or soybean oil which contain only 9% of this acid (22, 23). Furthermore, the fatty acid profile of the rapeseed oil used in this study as an example of a commonly consumed oil was typical (22).

In the present study, the calorie intake was significantly higher in rats fed the obesogenic diets, which led to the increase of the body weight and the epididymal fat mass by the end of the experiment (Table 3). The caloric value of the obesogenic diet was increased due to dietary lard, which was added to the diet in place of a proportion of complex carbohydrates, that is cellulose and corn starch. As a consequence of the lard addition, the content of SFAs and monounsaturated fatty acids (MUFAs) was also substantially increased in the obesogenic diet. On the other hand, the addition of strawberry seed oil to the basal and obesogenic diet increased the PUFA contents and decreased the MUFA contents compared to both rapeseed oil-containing diets. However, the aforementioned differences were more visible between the basal diets than the obesogenic diets due to the presence of lard in the later diets (Table 2). It especially concerns the dietary MUFA contents whose presence in lard is relatively high.

In this study, both the obesogenic diet and strawberry seed oil hindered the cecal SCFA production and negatively stimulated the cecal *β*-glucuronidase activity. SCFAs are the most important fermentation end products in the distal intestine and indirect nutrients for the body. They are readily absorbed from the distal intestine and have a role in the regulation of energy metabolism and many other metabolic features, like *de novo* lipogenesis (24, 25). In the present study, the decrease in butyrate concentration can be considered especially unfavorable because this SCFA appears to protect against colon carcinogenesis (26). On the other hand, an elevated activity of microbial *β*-glucuronidase in the distal intestine has been widely suggested as a risk factor for colorectal cancer (27). A probable explanation for the aforementioned changes in the cecal digesta is the high-fat diet-induced overflow of bile acids into the distal intestine, which has been recently proposed as the main reason affecting the gut microbiota (28). It was shown on rats that cecal *β*-glucuronidase activity is closely controlled by bile flow (29), whereas the ingestion of PUFAs is another important dietary factor, aside from a high content of dietary fat *per se*, that can intensify bile output and bile acid secretion (30, 31). This explains the considerable increase in *β*-glucuronidase activity noted in the current study in group O-SO. Interestingly, Islam et al. (32) showed that dietary supplementation with cholic acid leads to similar disorders in the cecum of rats to those induced by a high-fat diet, including a distinct decrease in SCFA production. Furthermore, the decrease in dry matter of the cecal digesta that was observed after feeding with the obesogenic diet could have been due to the lower proportion of dietary cellulose, which is known to be hardly fermented in the distal intestine. The results of a previous study performed in our laboratory indicate that dietary cellulose levels of 2.5–5% have no influence on the cecal SCFA contents in rats (19). However, in this study, the proportion of corn starch was also notably lower in the obesogenic diet compared to the basal diet. Thus, an additional explanation for the diminished SCFA production by the obesogenic dietary regimen may also be sought in the reduced consumption of resistant starch, which is known to be easily fermented in the cecum and to protect against colonic DNA damage (33).

The beneficial effects of dietary PUFAs on lipid metabolism are mainly attributed to their ability for the induction of fatty acid oxidation in the liver and skeletal muscles and simultaneous suppression of hepatic lipid synthesis (34). In the present study, both the obesogenic diet and strawberry seed oil decreased the SREBP-1c content of the liver (Fig. 2). SREBP-1c is one of the most important transcription factors that regulates the hepatic expression of enzymes involved in the *de novo* synthesis of fatty acids (35). In this study, the decrease in the SREBP-1c content *via* the obesogenic diet could have resulted from the decreased level of dietary carbohydrates, which were replaced with fat. This phenomenon might have initially reduced the level of postprandial insulin, which is recognized to be the main activator of SREBP-1c at both the transcriptional and the posttranslational levels (36). Dietary strawberry seed oil decreased the SREBP-1c content, likely due to its abundance in α-linolenic acid, which is able to suppress SREBP-1c expression, as was shown in recent *in vitro* and *in vivo* studies (37, 38). In the current study, the decrease in *de novo* lipogenesis *via* hepatic SREBP-1c was reflected by the lower plasma TG levels noted after the consumption of obesogenic diet and strawberry seed oil (Table 6). Additionally, the liver fat proportion was also considerably decreased by the strawberry seed oil (Table 5). However, there was no strict correlation between the SREBP-1c content and the lipid-lowering effects of strawberry seed oil. This suggests that another regulatory mechanisms, including other transcription factors (35), and other components of strawberry seed oil, like linoleic acid, phytosterols, and phenolics (14, 20), could have also played a role in the modulation of lipid metabolism in the present study. Conversely, the obesogenic dietary regimen increased the liver fat proportion, which was associated with a slight but significant increase in the plasma CRP concentration (Table 5). This finding indicates on a low-grade inflammation in the organism, which can be caused by the gut microbiota and is known to be involved in the development of fatty liver disease (39). Interestingly, in a toxicological study, Pieszka et al. (20) showed certain potential benefits of the redox status after strawberry seed oil ingestion for 5 weeks by rats. However, the authors did not find any favorable effects on blood lipids. An explanation of this discrepancy can be found in the methodological differences between the cited study and the present study. In the cited study, strawberry seed oil was administered to rats once daily by oral gavage at a dose of 0.8 mL and at the expense of a regular chow diet, which was also given to the control group (20). As a result, the overall consumption of fat and energy was increased in the strawberry seed oil-supplemented group, which was likely of crucial importance to the lipid metabolism of rats. Furthermore, in the present study, an important marker for cardiovascular health that considerably decreased due to the consumption of strawberry seed oil was the AI of plasma. In humans, the values of this index are inversely correlated with the lipoprotein particle size, thus predicting atherogenicity (18).

## Conclusion

Dietary strawberry seed oil rich in essential fatty acids can unfavorably affect metabolite formation in the distal intestine and putative indices related to colon cancer risk. On the other hand, the strawberry seed oil can ameliorate the lipid metabolism by decreasing liver fat accumulation, the plasma TG level and the AI of plasma compared to dietary rapeseed oil used in this study as an example of a commonly consumed oil with established nutritional quality. This potent lipid-lowering activity of strawberry seed oil is partly dependent on the suppression of de novo lipogenesis via hepatic SREBP-1c, whereas the disturbances in microbial metabolism seem to be associated with the overflow of bile acids into the distal intestine. Moreover, the observed effects are partly due to the synergistic action of dietary strawberry seed oil and the obesogenic diet, which suggests that a dietary supplement can disparately affect, beneficially or detrimentally, different body systems and the outcomes are partly dependent on the diet type.
